# Obesity and diabetes accelerate hepatocarcinogenesis via hepatocyte proliferation independent of NF-κB or Akt/mTORC1

**Published:** 2016-01-19

**Authors:** Evi Arfianti, Claire Z Larter, Seungsoo Lee, Vanessa Barn, Geoffrey Haigh, Matthew M. Yeh, George N. Ioannou, Narci C. Teoh, Geoffrey C. Farrell

**Affiliations:** 1 Liver Research Group, Australian National University Medical School, The Canberra Hospital, Australian Capital Territory, Australia; 2 Faculty of Medicine, Universitas Riau, Pekanbaru, Indonesia; 3 Division of Gastroenterology, University of Washington, Seattle, Washington, United States; 4 Department of Pathology, University of Washington, Seattle, Washington, United States

**Keywords:** ataxia-telangiectasia mutated, glutathione-S-transferase pi, rapamycin, interleukin-6, signal transducer and activator of transcription 3

## Abstract

**Background:**

There are strong links between obesity, diabetes and hepatocellular carcinoma (HCC), but molecular mechanisms remain unclear.

**Aim:**

We tested the proposed involvement of NF-κB, IL-6/STAT3 and Akt/mTORC1 before onset (at 3 months) and at onset (6 months) of accelerated hepatocarcinogenesis in DEN-injected obese and diabetic *foz/foz* compared to lean wildtype (*Wt*) mice, and also studied the hepatocyte proliferative response to DNA damage between the obese and lean lines.

**Methods:**

Male *foz/foz* and *Wt* littermates fed normal chow were DEN-injected (10mg/kg i.p.) at age 12-15 days. To test the effect of mTOR inhibitor on growth of dysplastic hepatocytes, a separate cohort of DEN-injected *foz/foz* mice was administered rapamycin (4 mg/kg body weight/day).

**Results:**

*foz/foz* mice developed obesity, hyperinsulinemia, diabetes, adipokine dysregulation and fatty liver, without increased serum or liver TNF-α or serum IL-6. All DEN-injected *foz/foz* mice developed HCC by 6 mths *vs*. 0/10 lean *Wt*. At 3 mths, there were more dysplastic hepatocytes in DEN-injected *foz/foz* than *Wt*, with increased liver injury (serum ALT), hepatocyte apoptosis (M30-positive cells) and proliferation (cyclin D1, cyclin E, PCNA), but neither NF-κB nor STAT3 activation. *foz/foz* livers exhibited upregulation of DNA damage sensors ATM and ATR, with inadequate cell cycle checkpoint controls (CHK1, CHK2, p53, p21). Akt and mTORC1 were highly activated in livers from *foz/foz vs. Wt* mice. Despite such activation, rapamycin failed to reduce growth of dysplastic hepatocytes.

**Conclusions:**

Accelerated DEN-induced HCC in obese/diabetic mice is linked to enhanced growth of dysplastic hepatocytes that cannot be attributed to NF-κB or IL-6/STAT3 activation, nor to sustained mTORC1 activation. The critical mechanism for obesity-enhanced hepatocarcinogenesis lies in the disconnection between hepatocellular injury with DNA damage, and an unrestrained proliferative response.

**Relevance for patients:**

This study supports the epidemiological data linking obesity, diabetes and fatty liver disease with increased risk for developing HCC. The findings also suggest that mTORC1 inhibition may not be beneficial in the prevention of obesity-related hepatocarcinogenesis.

## 1. Introduction

Obesity increases hepatocellular carcinoma (HCC) risk up to 4-fold [1-3], but the molecular pathways driving such promotion of hepatocarcinogenesis remain unclear. Park *et al*. [4] produced evidence that tumor necrosis factor-α (TNF-α) signaling to enhance IL-6 production and could be correlated with HCC development in obese mice. However, not all obese models show increases in pro-inflammatory cytokines [5,6]. Further, although abrogation of IL-6 signaling by knockout of IL-6 receptor α (IL-6Rα) prevents DEN-induced HCC in lean mice, *Il-6r* *α*^-/-^ mice fed a high-fat developed liver tumours to the same extent as *Wt* [7]. Thus, while IL-6-dependent signaling plays a role in diethylnitrosamine (DEN)-induced HCC in lean mice, obesity is more likely to promote hepatocarcinogenesis by a different mechanism.

Another potential link between obesity and HCC is hyperinsulinemia resulting from insulin resistance, which exerts growth effects either directly or via release of insulin-like growth factor-1 (IGF-1) [8,9]. In hepatocytes, protein kinase B (Akt) and mammalian target of rapamycin complex 1 (mTORC1) are important mediators of insulin action [10,11]; ~ 40-50% of HCCs demonstrate Akt activation, and/or mTOR activation [12,13]. A role for mTORC1 in hepatocarcinogenesis is further supported by findings in mice with liver-specific knockout of tuberous sclerosis protein 1 (*Tsc1*^-/-^). TSC1 constitutively suppresses mTORC1, and its inactivation leads to sustained mTORC1 activation [14]. mTORC1 activation also occurred in a dietary obesity model [4], but mTORC1 inhibition failed to suppress hepatocarcinogenesis. Instead, mTOR inhibition by rapamycin increased liver injury, IL-6 release and signal transducer and activator of transcription 3 (STAT3) activation [15].

In *Mdr2*^-/-^ mice, hepatocarcinogenesis is associated with activation of the DNA damage-response machinery that increases genomic instability [16], a feature of human HCC [17]. Earlier, we used mice defective for the non-homologous end joining pathway of DNA strand break repair, *Ku70*^-/-^ mice, to show how DEN injection caused chromosomal instability (CIN), with resultant loss of p53 function that facilitated accelerated onset of hepatocarcinogenesis [18]. Together, these findings indicate that cellular responses to DNA damage, an expected consequence of oxidative stress in non-alcoholic steatohepatitis (NASH) or cirrhosis, cause CIN, which in turn contributes to the multistep process of hepatocarcinogenesis. The present studies predicated that such a pathway may explain accelerated hepatocarcinogenesis in obesity and diabetes-related fatty liver disease.

To clarify the tumorigenic effects of obesity in hepatocarcinogenesis, we employed *foz/foz* mice, an obesity model in which key features of human metabolic obesity occur [19,20]: diabetes, metabolic syndrome and non-alcoholic fatty liver disease (NAFLD)/NASH. We first sought correlations between the rapid onset of HCC in obese *foz/foz* mice with serum cytokine changes and hepatocyte activation of NF-κB and STAT3 that others have suggested important. Having found no such associations, we clarified the strong associations between hyperinsulinemia and Akt/mTORC1 activation, then tested whether blockade of mTORC1 with rapamycin could slow onset of hepatocarcinogenesis. Finally, we characterized the DNA damage response in DEN-injected obese mice. This allowed us to identify defective signaling to cell cycle checkpoint regulators as the defect central to accelerated development of HCC in obese and diabetic mice.

## 2. Materials and Methods

### 2.1. Animals

Male *Alms1* mutant (*foz/foz*) NOD.B10 mice and wild type (*Wt*) littermates were injected intraperitoneally (i.p) with DEN (10 mg/kg); saline to controls at 12-15 days of age (n = 11-12 mice/group). From weaning, they were fed chow diet (Specialty Feeds, Glen Forrest, Australia) to the times indicated in figure legends. All animal experiments were approved by the Australian National University Animal Ethics Committee (protocol A2011/40).

### 2.2. Rapamycin in vivo study

DEN-injected male *foz/foz* mice were fed a chow diet with or without rapamycin (4 mg/kg body weight/day, LC Laboratories, Woburn, MA, USA) (n = 9-10 mice/group) to 3 mths of age. Two weeks before sacrifice, glucose tolerance was measured after intraperitoneal glucose injection (2 g/kg body weight).

### 2.3. Serum and hepatic lipid analyses

Serum biochemistry was measured using automated techniques (ACT Pathology, the Canberra Hospital). Serum insulin (Millipore, Billerica, MA, USA), leptin, adiponectin, IL-6, TNF-α, IGF-1 and IGF-BP3 (R&D systems, Minneapolis, MN, USA) were measured by enzyme-linked immunosorbent assay (ELISA). Hepatic triglycerides and cholesterol ester were quantified using high-performance liquid chromatography (HPLC) as reported [21] and results were normalized to wet liver weight (g).

### 2.4. Liver histology and immunohistochemistry

Formalin-fixed, paraffin-embedded liver sections (4 µm) were stained with hematoxylin and eosin (H&E). Histological diagnosis for HCC was performed blindly by an experienced liver pathologist. Dysplastic hepatocytes were visualized by glutathione-S-transferase pi (GST-pi, gift from Philip Board, John Curtin School of Medical Research, Australia), immunohistochemistry (IHC), hepatocyte apoptosis by M30 (cytokeratin-18 [CK-18]-fragmentation peptide) IHC, and proliferation by proliferating cell nuclear antigen (PCNA) IHC, as described [18].

### 2.5. Analysis of hepatic genes and proteins

Gene and protein expression were assayed using semi-quantitative real time PCR and immunoblotting, respectively as previously reported [22]. Primer sequences and antibody conditions will be supplied upon request.

### 2.6. Statistical Analyses

Data (mean ± SEM) were analyzed by one-way or two-way analysis of variance (ANOVA) followed by *post-hoc* analysis using Bonferroni’s multiple comparison test. Statistical analyses were performed using GraphPad Prism 6.02 (GraphPad Software, CA, USA). *P* < 0.05 was considered significant.

## 3. Results

### 3.1. DEN-induced hepatocarcinogenesis is accelerated in foz/ foz mice in association with metabolic complications of obesity

**Figure 1.**
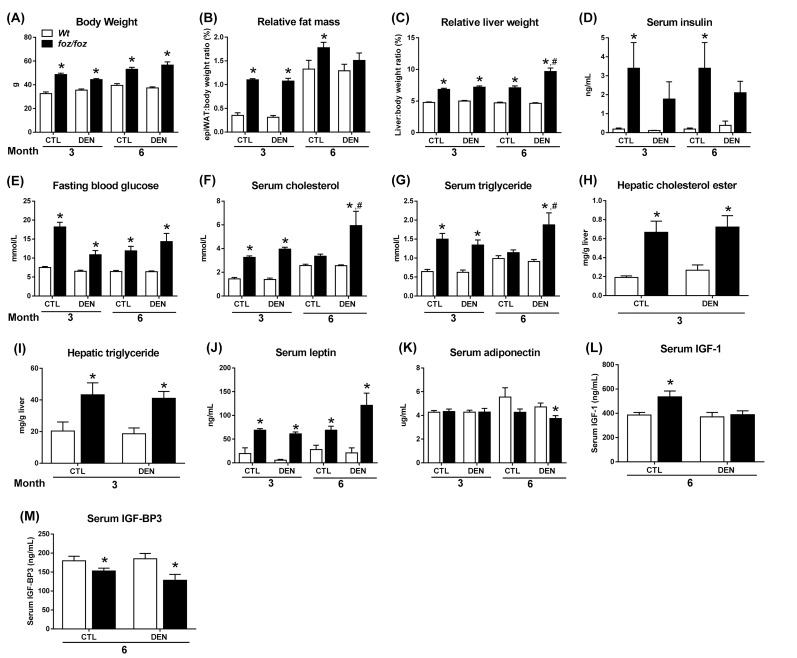
Body weight, tissue weights, and metabolic parameters in *foz/foz* and *Wt* mice. (A) Body weight, (B) relative epididymal white adipose tissue (epiWAT) mass and (C) relative liver weight in saline (CTL) and DEN-injected *foz/foz* and *Wt* mice. (D) Serum insulin was quantified by enzyme-linked immunosorbent assay (ELISA) and (E) blood glucose was determined after fasting for 4 hrs. Serum (F) cholesterol and (G) triglyceride in *foz/foz* and *Wt* mice were measured using automated techniques (see Materials and Methods). Hepatic (H) cholesterol esters and (I) triglycerides levels at 3 mths were determined by high-performance liquid chromatography. Serum (J) leptin, (K) adiponectin, (L) IGF-1 and (M) IGF-BP3 were measured by ELISA. Data are mean ± SEM (n = 9-15) mice/group. ^*^P < 0.05, *vs*. treatment-matched, genotype control, ^#^*P* < 0.05, *vs*. genotype-matched, treatment control, by one way or two-way ANOVA with Bonferroni’s post hoc test.

**Figure 2.**
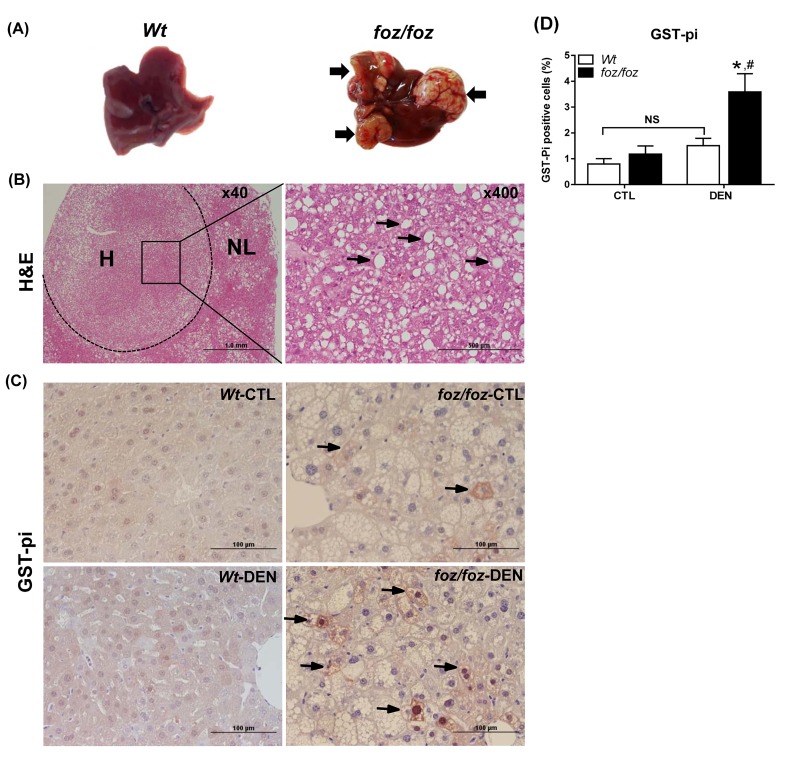
*foz/foz* mice display steatohepatitic HCC at 6 mths and increased number of dysplastic hepatocytes at 3 mths compared to *Wt* mice. (A) Gross appearance of livers from DEN-injected *foz/foz* and *Wt* mice at 6 mths. Arrows point to some of the tumours. (B) Representative H&E stained liver sections from a DEN-injected *foz/foz* mouse show HCC (H) and surrounding non-tumorous liver (NL) (x40 magnification), and a typical steatohepatitic HCC (x400 magnification). Arrows point to macrovesicular steatosis. (C) GST-pi IHC in liver sections from 3-mth-old *foz/foz* and *Wt* mice was used to determine dysplastic hepatocytes. Arrows indicate GST-pi-positive hepatocytes (x400 magnification). (D) Quantification of GST-pi-positive cells (see Materials and methods). Data are mean ± SEM from 9-10 mice/group. ^*^P < 0.05, *vs*. treatment-matched, genotype control, ^#^*P* < 0.05, *vs*. genotype-matched, treatment control.

As reported [19,22]*, foz/foz* mice were heavier than *Wt* at 3 and 6 mths (Figure 1A), with increased adiposity (peri-epi-didymal white adipose tissue (WAT) mass; Figure 1B) and hepatomegaly (Figure 1C). At 3 mths, there were no macroscopic liver nodules in DEN-injected *foz/foz* or *Wt* mice (Table 1), but all *foz/foz* mice developed HCC by 6 mths (Table 1; Figure 2A), with two mice bearing lung metastases. No *Wt* (lean) littermates had macroscopic liver tumours at this time (Table 1; Figure 2A). As shown in Figure 2B, histological examination of these tumors showed features described as the steatohepatitic variant of HCC, with large- droplet steatosis and mild inflammatory cell infiltration [23]. Relative liver weight was greater in DEN-injected obese mice at 6 mths (Figure 1C), which was attributable to large tumour burden (Figure 2A). In contrast, relative liver weight of *Wt* mice was unchanged (Figure 1C), consistent with only 1-2 pin point nodules observed in DEN-injected *Wt* mice at 6 mths (data not shown). At 9 mths, all mice developed HCC but lung metastases were more frequent in *foz/foz* mice, consistent with the earlier onset and more aggressive nature of HCC in obese/diabetic mice (Table 1). Since the focus of the present studies was on the molecular pathways that *precede* onset of obesity-related HCC, subsequent measurements were performed on tissues harvested at 3 and/or 6 mths.

*foz/foz* mice exhibited hyperleptinemia (Figure 1J), while serum adiponectin was lower than *Wt* only in 6-mth-old DEN-treated *foz/foz* mice (Figure 1K). At both 3 and 6 mths, serum insulin was higher in *foz/foz* than *Wt*, albeit the apparent difference was not significant in DEN-treated mice (Figure 1D). Fasting blood glucose (FBG) was also increased (Figure 1E), all *foz/foz* mice developed diabetes (FBG > 8 mml/L), and serum cholesterol and triglyceride were higher (Figure 1F,G). DEN injection exacerbated these changes in *foz/foz* but not *Wt* mice. Consistent with the metabolic changes, livers of *foz/foz vs. Wt* mice at 3 mths showed increased hepatic cholesterol ester and triglyceride content (Figure 1H,I).

### 3.2. Enhanced growth of dysplastic hepatocytes in obese, diabetic foz/foz mice is associated with increased liver injury and hepatocellular proliferation

**Figure 3.**
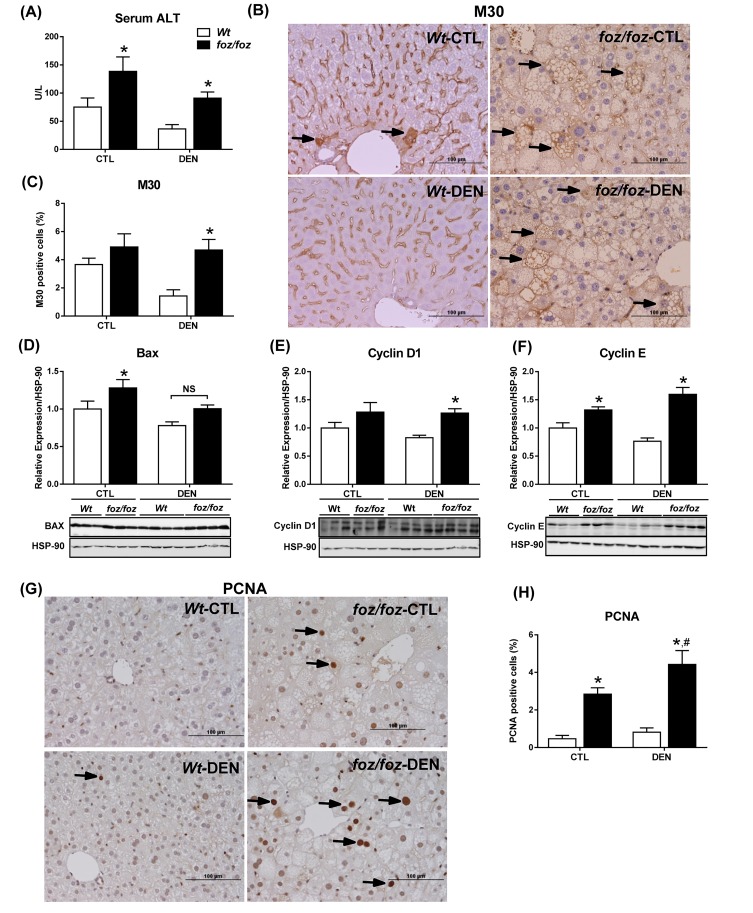
*foz/foz* mice exhibit increased hepatocyte injury, apoptosis and proliferation compared to *Wt* mice at 3 mths. (A) Serum alanine amino transferase (ALT) in *foz/foz* and *Wt* mice at 3 mths. (B) Cytokeratin-18 fragmentation (M30) immunostaining was used to determine hepatocellular cell death in livers from *foz/foz* and *Wt* mice. (C) Quantification (ImageJ) of M30-positive hepatocytes in *foz/foz* and *Wt* mice. Hepatic expression of (D) pro-apoptotic Bax, (E) cyclin D1 and (F) cyclin E were determined using immunoblotting. HSP-90 was used as a loading control. (G) PCNA immunostaining of liver sections from *foz/foz* and *Wt* mice was used to (H) quantify hepatocytes in cell cycle. *Arrows* indicate positive staining (x400 magnification). Data are mean ± SEM from 9-11 mice/group. ^*^P < 0.05, *vs*. treatment-matched, genotype control, ^#^*P* < 0.05, *vs*. genotype-matched, treatment control.

At 3 mths, saline-injected *foz/foz* mice exhibited a small number of GST-pi-positive hepatocytes. DEN injection significantly increased the number of GST-pi-positive hepatocytes in *foz/foz* mice, but not in corresponding *Wt* littermates (3.7 ± 0.6% *vs*. 1.6 ± 0.2 %, Figure 2C,D). Irrespective of DEN treatment, serum ALT was higher in *foz/foz* mice than *Wt* (Figure 3A), as was hepatocyte apoptosis by M30-immunostaining (Figure 3B,C), and pro-apoptotic Bax expression (Figure 3D). Persistently increased hepatocyte injury incites compensatory hepatocellular proliferation so as to maintain organ function. Consistent with this, hepatic expression of cyclin D1 and cyclin E were upregulated in *foz/foz* compared to *Wt* at 3 mths (Figure 3E,F), and there were abundant PCNA-positive cells in livers from obese compared to lean mice (Figure 3G,H).

**Table 1.** HCC incidence and frequency of lung metastases in *foz/foz* and *Wt* mice

Genotype	3 mths	6 mths		9 mths	
	HCC	HCC	Metastases	HCC	Metastases
*Wt*	0	0/11	–	11/11 (100%)	1/11 (9.1%)
*foz/foz*	0	12/12 (100%)	2/12 (17%)	11/11 (100%)	7/11 (64%)

Abbreviations: mths, months; HCC, hepatocellular carcinoma; *Wt*, wild-type.

### 3.3. ATM and ATR are induced in livers from foz/foz mice, but p53 and p21 fail to halt proliferation of damaged hepatocytes

**Figure 4.**
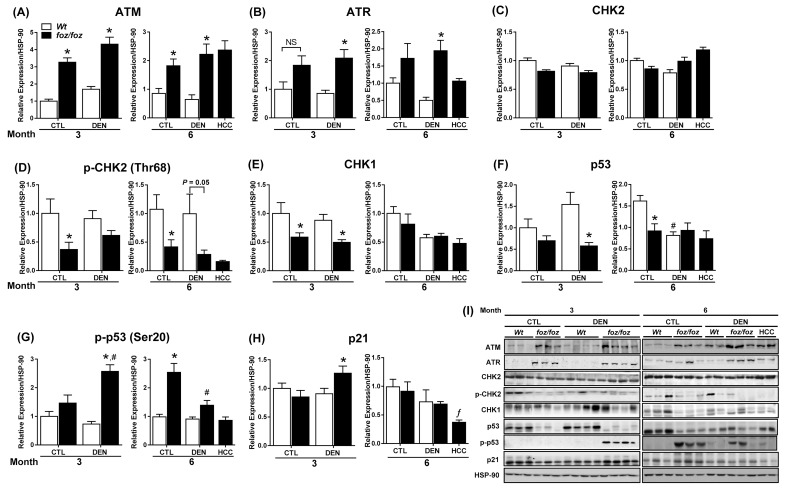
Differential up-regulation of ATM, ATR, and target proteins that are cell cycle regulators in *foz/foz* and *Wt* mice. Hepatic expression of (A) ATM, (B) ATR, (C) CHK2, (D) CHK2 phosphorylation, (E) CHK1, (F) total p53, (G) p53 Ser20 phosphorylation and (H) p21 in *foz/foz* and *Wt* mice at 3 and 6 mths were determined by immunoblotting. (I) Representative Western Blots for ATM, ATR, CHK2, p-CHK2, CHK1, p53, p-p53, p21 and HSP-90 (as loading control). Data are mean ± SEM from 10-12 mice/group. ^*^P<0.05, *vs*. treatment-matched, genotype control, ^#^*P*<0.05, *vs*. genotype-matched, treatment control.

In the presence of DNA damage, cells sense DNA strand breaks via ataxia-telangiectasia mutated (ATM) and ataxia-telangiectasia Rad-3 related (ATR) proteins. In turn, these sensors coordinate cellular responses to DNA lesioning, such as induction of cell cycle checkpoint proteins that inhibit the proliferation of damaged, preneoplastic hepatocytes [24, 25]. Hepatic ATM expression increased markedly in *foz/foz* mice compared with *Wt*, regardless of DEN (Figure 4A). ATR was also significantly induced in livers from DEN-injected *foz/foz* (*vs. Wt*) at both times (Figure 4B). While total CHK2 did not differ across groups (Figure 4C) at 6 mths, phosphorylated CHK2 was lower in livers from *foz/foz* mice (and in HCC) than *Wt* after DEN (Figure 4D). At 3 mths, hepatic CHK1 expression was also lower in obese *foz/foz* than in lean *Wt* littermates (irrespective of DEN injection), but values were comparable at 6 mths (Figure 4E).

p53 is modified post-translationally by phosphorylation at multiple sites, some of which activate p53 function in response to DNA damage [26]. At 3 mths, p53 expression levels were substantially reduced in livers from DEN-injected *foz/foz* than *Wt* mice (Figure 4F), but at 6 mths values were similar in the two lines. On the other hand, p53 Ser20 phosphorylation increased in livers from DEN-injected *foz/foz vs. Wt* animal at 3 mths, consistent with ATM induction (Figure 4G), but by 6 mths there was markedly less p53 phosphorylation in livers from DEN-injected *foz/foz* mice. As a result, p21, although upregulated at 3 mths in livers from *foz/foz* mice after DEN injection (*vs. Wt* littermates), was markedly decreased in HCC *vs*. dysplastic livers (Figure 4H).

### 3.4. NF-κB and STAT3 are not activated in livers from obese foz/foz mice

**Figure 5.**
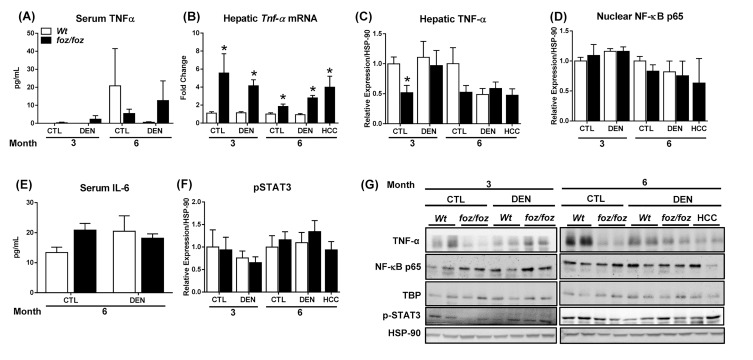
Despite increases in hepatic *Tnf-* *α* mRNA in *foz/foz vs. Wt* mice, serum IL-6, activation of NF-κB and STAT3 phosphorylation do not differ. (A) Serum TNF-α was measured by enzyme-linked immunosorbent assay. (B) TNF-α mRNA was determined by semi-quantitative real time polymerase chain reaction in livers from *foz/foz* and *Wt* mice. Hepatic expression of (C) TNF-α (whole lysates) and (D) NF-κB p65 (nuclear extracts) were examined by immunoblotting. (E) Serum IL-6 levels were measured by enzyme-linked immunosorbent assay in *foz/foz* and *Wt* at 6 mths. (D) Phospho (p)-STAT3 expression in livers from *foz/foz* and *Wt* mice was assessed by immunoblotting. (G) Representative Western Blots for TNF-α, NF-κB p65, p-STAT3, as well as TATA-binding protein (TBP) and heat shock protein (HSP)-90 (as respective loading controls for nuclear and whole liver extracts). Data are mean ± SEM from 7-10 mice/group. ^*^P < 0.05, *vs*. treatment-matched, genotype control.

There was no increase in serum TNF-α in obese *foz/foz* mice; values were highly variable, being detected in < 10% *foz/foz* and *Wt* mice, and there was no correlation with HCC (Figure 5A). Within liver tissue, hepatic transcript levels of *Tnf-* *α* were persistently upregulated in obesity and HCC (Figure 5B), but this did not translate into increased expression of hepatic TNF-α (Figure 5C,G). Conversely, saline-injected *foz/foz* mice exhibited lower hepatic TNF-α compared to *Wt* counterparts at 3 mths. Accordingly, hepatic nuclear NF-κB p65 did not differ between obese *foz/foz* and lean *Wt* mice (Figure 5D,G). Levels of serum IL-6 (which is induced by TNF-α) were also comparable across all groups (Figure 5E). IL-6 has been implicated via the activation of Janus kinase (JAK) 2/STAT 3 pathway in hepatocarcinogenesis [4,27]. In the present work, consistent with the failure of serum IL-6 to increase, there was no activation of STAT3 in fatty livers of these obese diabetic mice (Figure 5F,G).

### 3.5. mTORC1 signaling is activated in livers and HCCs from obese mice

**Figure 6.**
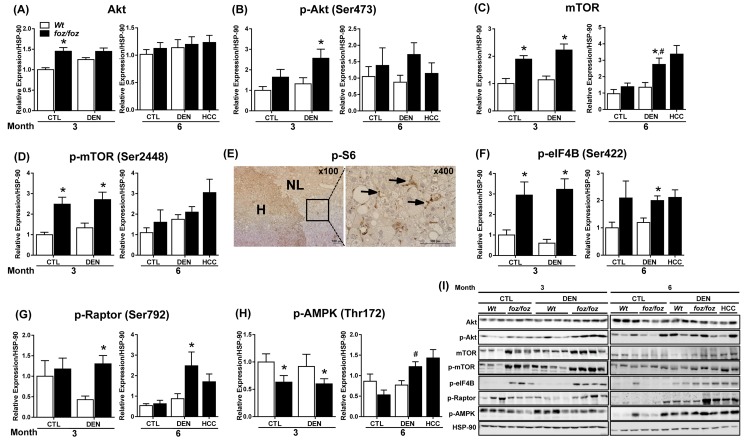
Activation of Akt/mTORC1 signaling cascade occurs in livers and HCCs from *foz/foz* mice. Hepatic expression of (A) total Akt, (B) Akt phosphorylation, (C) total mTOR, (D) mTOR phosphorylation in *foz/foz* and *Wt* mice at 3 and 6 mths were examined by immunoblotting. (E) Representative phospho (p)-S6 immunostaining in HCC tissue (H) *vs*. surrounding non-tumorous liver (NL) from DEN-injected *foz/foz* mice at 6 mths. p-S6 positive staining localized to non-parenchymal cells and lipid-laden non-malignant hepatocytes (Arrows). Hepatic expression of (F) p-eIF4B, (G) p-raptor and (H) p-AMPK was determined by immunoblotting. (I) Representative Western blots for Akt, p-Akt, mTOR, p-mTOR, p-eIF4B, p-Raptor, p-AMPK and HSP-90 (as loading control). Data are mean ± SEM from 6-11 mice/group. ^*^P < 0.05, *vs*. treatment-matched, genotype control, ^#^*P* < 0.05, *vs*. genotype-matched, treatment control.

In contrast to the lack of correlation with serum leptin, TNF-α, IL-6, NF-κB and STAT3 activation, hyperinsulinemia (Figure 1D) in the *foz/foz* model remained a candidate enhancer of hepatocarcinogenesis [8,9]. In the present study, serum IGF-1 was higher, and serum IGF-binding protein (IGF-BP) 3 decreased in obese *foz/foz* compared to lean *Wt*, irrespective of DEN (Figure 1L,M). These changes are consistent with increased bioavailability of IGF-1. We therefore examined metabolic pathways known to be affected by hyperinsulinemia/IGF-1. At 3 but not 6 mths, total Akt was induced in livers from saline-injected obese *foz/foz* compared to lean *Wt* animals (Figure 6A). Likewise, Akt phosphorylation increased in livers from DEN-treated obese *foz/foz* mice at 3 but not 6 mths (Figure 6B). Akt activation can activate the master “switch” of growth regulation, mTOR. At 3 mths, mTOR was upregulated (Figure 6C) and mTOR phosphorylation increased in livers of obese *foz/foz* compared to lean *Wt* mice, irrespective of DEN. Such mTOR activation was no longer evident at 6 mths (Figure 6D).

To establish whether mTOR activation reflected mTORC1 signaling, we examined known downstream targets [28]. As determined by IHC, we demonstrated an increased phosphorylation of ribosomal S6 (mediated by p70S6 kinase 1) in HCC arising in *foz/foz* mice relative to surrounding non-tumorous livers (Figure 6E). mTORC1 also regulates eukaryotic initiation factor 4B (eIF4B), an important regulator of protein translation [29]. At 3 mths, livers from *foz/foz* mice exhibited enhanced eIF4B phosphorylation compared to *Wt*, irrespective of DEN (Figure 6F). At 6 mths, DEN increased phosphorylation of eIF4B in livers from *foz/foz* but not *Wt*. Activation of these mTOR targets is consistent with the observed increase in phosphorylation of signaling intermediate raptor in livers from *foz/foz* but not *Wt* mice after DEN (Figure 6G). The AMP-activated protein kinase (AMPK), the main sensor of cellular energy status, inhibits mTORC1 [30,31]. Consistent with mTORC1 activation, AMPK phosphorylation decreased in livers from *foz/foz* compared to *Wt* mice (regardless of DEN) at 3 mths (Figure 6H), but by 6 mths AMPK was actually enhanced.

### 3.6. Rapamycin fails to inhibit growth and transformation of dysplastic hepatocytes in obese mice

**Figure 7.**
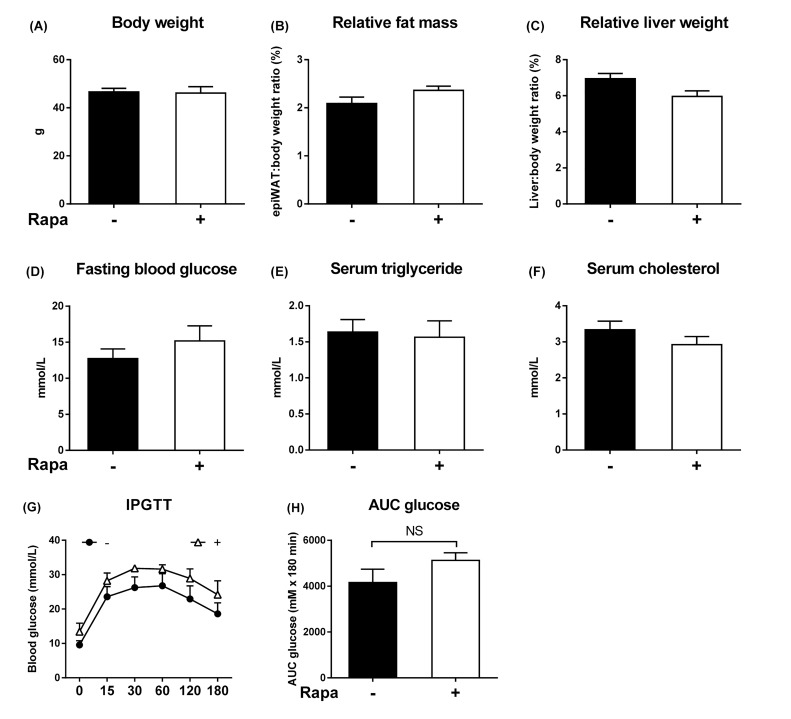
Lack of effect of rapamycin on body weight, adiposity, hepatomegaly, dyslipidemia, and glucose metabolism in *foz/foz* mice. Rapamycin feeding (4 mg/kg body weight/day) did not affect (A) body weight, (B) adiposity, (C) hepatomegaly, (D) fasting blood glucose levels, serum (E) triglyceride, and (F) cholesterol in *foz/foz* mice at 3 mths. (G-H) The apparent slight impairment of glucose tolerance after rapamycin exposure was not significant. Data are mean ± SEM (n = 9-10/group).

**Figure 8.**
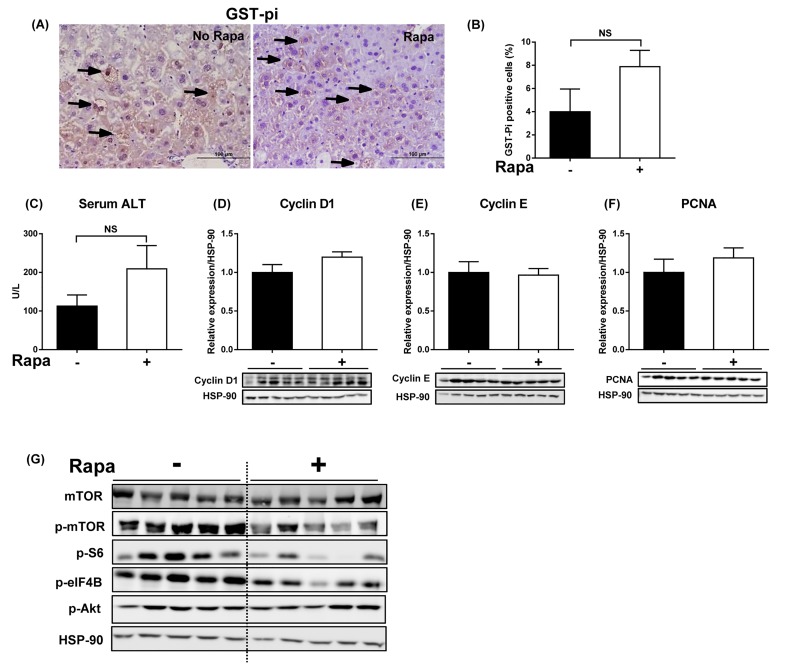
Rapamycin administration in *foz/foz* mice suppresses activation of hepatic mTORC1-eIF4B, but fails to inhibit growth of dysplastic hepatocytes. DEN-injected *foz/foz* mice were fed with or without rapamycin (4 mg/kg body weight/day) until 3 mths of age. (A) Representative images of GST-pi-stained liver sections and (B) GST-pi quantification demonstrated a trend towards increased numbers of GST-pi positive cells after rapamycin administration in *foz/foz* mice. (C) Serum ALT levels appeared higher (NS) in rapamycin-fed mice, while (D) cyclin D1, (E) cyclin E, and (F) PCNA remained unaltered. Data are mean ± SEM from 9-10 mice/group. (G) Effects of rapamycin administration on Akt/mTORC1 signaling proteins were analyzed by immunoblotting.

If mTORC1 is a critical node for progression to HCC, inhibition of mTOR signaling should delay hepatocarcinogenesis. Chronic rapamycin intake failed to alter body and tissue weights in *foz/foz* mice, and there was no change in hepatomegaly (Figure 7A-C). Similarly, the metabolic abnormalities, including glucose tolerance, were neither ameliorated nor exacerbated these changes significantly (Figure 7D-H). Despite the evidence of mTORC1 activation early in hepatocarcinogenesis in *foz/foz* mice, rapamycin administration failed to reduce the number of dysplastic hepatocytes at 3 mths (Figure 8A,B). In line with a recent study [15], chronic inhibition of mTORC1 signaling by rapamycin tended to increase serum ALT levels compared to untreated *foz/foz* mice (Figure 8C), but markers of hepatocyte proliferation were not altered (Figure 8D-F). As anticipated, rapamycin supplementation decreased mTOR phosphorylation, caused a slight reduction (NS) of S6 phosphorylation and decrease of eIF4B phosphorylation, but there was no change in Akt phosphorylation(Figure 8G).

## 4. Discussion

In the present study, we demonstrated that obese, diabetic *foz/foz* mice exhibit early onset (6 mths) DEN-induced HCC, whereas lean *Wt* mice do not develop HCC until 9 mths, a time course consistent with previous studies [18,32]. The earlier onset and more aggressive nature of DEN-induced HCC with obesity and diabetes were supported by the high rate of pulmonary metastases at 9 mths, 60% in obese *vs*. 10% in lean *Wt* mice. Others have used genetic (leptin or leptin receptor defective) and dietary models to show that obesity enhances DEN-induced hepatocarcinogenesis [4,33,34]. In *foz/foz* mice, such accelerated onset of HCC is associated with hyperinsulinemia, diabetes, hyperleptinemia, hypoadiponectinemia and fatty liver, all relevant to the metabolic complications of human obesity.

In the present studies, enhanced growth of dysplastic hepatocytes preceded development of HCC in obese *foz/foz* mice. Similar precancerous lesions are present in cirrhosis and/or chronically injured liver [35], and indicate the first change in the multistep process of hepatocarcinogenesis both in humans [17,36,37], and in DEN-injected mice [18,38-40]. In *foz/foz* mice, obesity appears to promote dysplastic change even in the absence of carcinogen, and with DEN, the number of dysplastic cells greatly exceeded that of lean mice. Enhanced growth of dysplastic hepatocytes in *foz/foz* mice was associated with more severe liver injury (serum ALT) and apoptosis, as well as increased proliferative activity, indicated by cyclin D1, E, and PCNA expression. Compensatory hepatocellular proliferation, an expected response to persistent hepatocyte cell death, contributes mechanistically to hepatocarcinogenesis animal models [41,42], and is also present in hepatitis C-cirrhosis before onset of human HCC [43].

Another striking and novel finding here is that DNA damage sensors (ATM and ATR) are upregulated in livers from obese compared to lean mice, even without DEN administration. However, such up-regulation failed to activate CHK1 or CHK2. CHK2 regulates stabilization and transcriptional activation of p53. Lack of CHK2 could at least partly explain our observation of low p53 expression in livers of obese mice. Consistent with defective transcriptional activation by p53 [44], p21, a critical cell cycle inhibitor, was upregulated during the early stage of hepatocarcinogenesis, but decreased in HCC compared to non-tumorous liver. These findings are consistent with the proposal that defective cell cycle checkpoint control exerted by p53 may be lost in obesity, and such loss could be pivotal to enhanced hepatocarcinogenesis.

A strength of the present work is the opportunity to examine the pre-malignant phase of hepatocarcinogenesis, allowing us to demonstrate alterations of molecular signaling that characterize the early development of obesity-associated HCC. At this stage, we found little evidence to support a key role of inflammation in obesity-related hepatocarcinogenesis. IL-6 was not changed, STAT3 was not activated, and hepatic TNF-α signaling was unlikely involved as NF-κB was also not activated. Instead, Akt and nutrient-sensing mTORC1 were activated early in fatty livers from obese and diabetic mice, attributable to hyperinsulinemia and increased circulating and free IGF-1. mTORC1 activates S6 and eIF4B, key regulators of cell growth that could promote growth of altered hepatocytes. The impaired activation of AMPK in livers from obese mice could further contribute to chronic activation of mTORC1 signaling by withdrawal of AMPK-mediated suppression. However, despite the capacity for persistent mTORC1 activation to facilitate growth and survival of altered cells, inhibition of mTORC1 with rapamycin over 3 mths failed to prevent growth of dysplastic hepatocytes. During the conduct of these experiments, Umemura *et al*. reported a similar finding [15]. Among potential explanations, increased liver injury in mice treated with rapamycin was also found in the present work.

In summary, the present studies using a mouse model that recapitulates all the metabolic complications of human obesity confirm that “metabolic obesity” enhances DEN-induced hepatocarcinogenesis. Onset of HCC was preceded by hepatocellular injury, resulting in apoptosis and compensatory hepatocellular proliferation, with increased survival and growth of dysplastic hepatocytes. It is evident, however, that mTORC1 is unlikely to be the critical pathway by which obesity and diabetes enhance development of HCC. The potential mechanism linking obesity to accelerated HCC is inadequate cell cycle checkpoint control by CHK2 and CHK1 in response to the increased DNA damage that occurs in fatty liver of obese/diabetic mice. This could affect the ability of p53 to inhibit proliferation of damaged and altered hepatocytes during the progression of dysplastic hepatocytes to HCC in obese mice. Tumor suppressor p53, and specifically lack of its appropriate function, is an “old player” in development of liver cancer, but it may still be a prime player in the link to obesity and metabolic liver disease.
